# Supplementary material for: Associated Factors of High Sedative Requirements within Patients with Moderate to Severe COVID-19 ARDS
Source: J Clin Med. 2022 Jan 25;11(3):588. doi: 10.3390/jcm11030588 (PMC8837042; doi:10.3390/jcm11030588)
Supplement: Supplementary file 1 [file jcm-11-00588-s001.zip › jcm-1503886-supplementary.pdf]

Supplement I

Sedation protocol	
**Expected short-term sedation:**	
Propofol	(continuously supplied as mg/kg/h)
└> if insufficient sedation level:	
- combination with dexmedetomidin	(continuously supplied as µg/kg/h)
└> if insufficient sedation level:	
- combination with lormetazepam	(continuously supplied as µg/kg/min)
Supplementary analgesia: Remifentanil	(continuously supplied as µg/kg/min)
**Expected long-term sedation:**	
≤ day 6* Propofol	(continuously supplied as mg/kg/min)
> day 7 switch to midazolam	(continuously supplied as mg/kg/h)
└> if insufficient sedation level:	
- combination with clonidine	(continuously supplied as µg/kg/h)
└> if insufficient sedation level:	
- combination with Esketamine	(continuously supplied as mg/kg/h)
Supplementary analgesia: Sufentanil	(continuously supplied as µg/kg/h)

* In line with recommendations to avoid propofol infusion syndrome [4,59].

Reference

4. Taskforce, D.A.S.; Baron, R.; Binder, A.; Biniek, R.; Braune, S.; Buerkle, H.; Dall, P.; Demirakca, S.; Eckardt, R.; Eggers, V.; et al. Evidence and consensus based guideline for the management of delirium, analgesia, and sedation in intensive care medicine. Revision 2015 (DAS-Guideline 2015)-short version. *Ger. Med. Sci.* **2015**, *13*, Doc19. https://doi.org/10.3205/000223.
59. Hemphill, S.; McMenamin, L.; Bellamy, M.C.; Hopkins, P.M. Propofol infusion syndrome: a structured literature review and analysis of published case reports. *Br. J. Anaesth.* **2019**, *122*, 448–459, https://doi.org/10.1016/j.bja.2018.12.025.
